# How science gets by with a little help from the Beatles: Detecting cultural wordplay in scientific literature using large language models

**DOI:** 10.1371/journal.pone.0356147

**Published:** 2026-09-09

**Authors:** Gabriel Budel, Robert E. Kooij, Michiel Tjepkema

**Affiliations:** 1 Faculty of Electrical Engineering, Mathematics and Computer Science, Delft University of Technology, Delft, The Netherlands; 2 Koninklijke KPN N.V., Rotterdam, The Netherlands; 3 Unit ICT, Strategy & Policy, TNO (Netherlands Organisation for Applied Scientific Research), The Hague, The Netherlands; 4 Faculty of Law, Radboud Universiteit Nijmegen, Nijmegen, The Netherlands; 5 Co-host to the Dutch Beatles podcast Fab4Cast, Hilversum, The Netherlands; Universidade Federal do Tocantins, BRAZIL

## Abstract

The Beatles’ cultural influence reaches well beyond music into television, fashion, and global media. As we show, it reaches into scientific publishing too. This study documents how often Beatles song titles and lyrics appear in academic article titles, identifying 2,237 exact references and 963 instances of creative wordplay in data from Scopus. Working from a curated list of Beatles songs and lyrics, we combine automated title queries with large-language-model screening, followed by manual verification, to detect both exact references and wordplay. To our knowledge, this is the first study to systematically identify wordplay on Beatles song titles and lyrics in scientific article titles. We report which titles and lyrics are referenced most often, trace how their use has grown over the decades, and show that this growth has outpaced the expansion of the scientific literature as a whole while varying systematically across disciplines. The result is the first large-scale, cross-disciplinary account of how the Beatles have left their mark on scientific writing.

## Introduction

Scientific writing and popular culture are more intertwined than they might first appear. Researchers routinely draw on cultural touchstones to frame their work: iconic phrases become metaphors, song titles appear in article titles, and widely recognized references help authors connect with broader audiences. Popular music in particular has left a distinct mark on academic publishing—from Bob Dylan’s lyrics embedded in biomedical papers [1] to the Clash’s ‘Should I Stay or Should I Go?’ repurposed across hundreds of scientific titles [2]. Of all musical artists to have influenced scientific communication in this way, none has done so as pervasively as the Beatles.

To appreciate why, it helps to understand the extraordinary breadth of the Beatles’ cultural reach. Their influence on popular culture manifests itself in a wide variety of ways. Beyond the obvious (namely their impact on the music of other artists), the Beatles are widely recognized as a major force in shaping popular culture. This article could easily have focused on their influence on TV series, films, or fashion, as some of their most famous phrases have lent themselves to TV programs in many countries. In the Netherlands, well-known TV programs focusing on romantic relationships and emotional encounters include ‘All You Need Is Love’ and ‘Hello, Goodbye’; the latter also airs in many other countries under the same title. Other references to the Beatles include *Lady Lazarus*; *Mad Men*, Season 5 (reference to ‘Tomorrow Never Knows’); *Bubbikins*; *The Crown*, Season 3 (multiple references to the Beatles); *Glee*, Season 5 (the season opener is a Beatles tribute). The cultural impact of the Beatles extends far beyond the numerous movies and documentaries explicitly dedicated to them and their music, such as the film *Yesterday* (2019), Peter Jackson’s nearly nine-hour *Get Back* (2021), or the documentary *Beatles ’64* (2024). More than 56 years after their breakup, the Beatles remain one of the most beloved cultural phenomena of all time, more so than any other band or artist.

As both scientists and Beatles fans, we noticed that their influence has also permeated the world of science. A search on the website of Nature reveals 116 articles in the Nature portfolio that explicitly contain the literal phrase ‘the Beatles’ in the article text (as of July 27, 2025). This illustrates that researchers frequently draw parallels between the subjects of their research and the work of the Beatles. For example, the band has even lent its name to a recognized challenge in drug development: the so-called “better than the Beatles problem”, which captures the difficulty of bringing to market a new drug whose benefits surpass those of the highly effective treatments already available [3,4]. The intersection of the Beatles and science is often presented with humor, too: a NASA research project on so-called *pingos* (ice-cored hills) was named *Pingo STARR*, an acronym for SubTerranean Aquifer Reconnaissance and Reconstruction [5].

These are just a few examples of how the Beatles have spread through the scientific world. This traffic between science and culture is not one-way: scientific progress has itself shaped culture (most famously giving rise to science fiction, which has in turn inspired real scientific advances [6,7]), making the exchange a mutual, even cyclical, one. In this article, we focus specifically on the use of Beatles song titles and lyrics in the titles of scientific publications, and we can safely say that the Beatles are the band most frequently referenced in scientific article titles. The reasons for referencing popular music in scientific titles vary. Sometimes, an otherwise technical or dry subject can be made more engaging by alluding to a well-known song, which can help in the competitive ‘publish or perish’ culture of academia. More broadly, bibliometric frameworks extending Kuhn’s model of scientific evolution observe that the usage of plain language and “easy-to-remember labels” serves as a vital prerequisite for a new idea to gain community traction [8,9]. This suggests that the appeal of culturally resonant titles goes beyond competitive signaling alone, playing a structural role in the popularization of scientific writing. A catchy title may attract more readers and, potentially, more citations [2]. This explains the title of our own article: With a little help from the Beatles, research papers may attract greater attention.

This is not the first study to investigate the use of Beatles song titles in scientific literature. Previous studies have examined Beatles references specifically in medical publications [10] or biomedical literature [11], often alongside references to other artists such as Bob Dylan. Our study differs in two important ways: first, it does not limit itself to one specific scientific discipline. Instead, we use two of the most comprehensive academic databases—Scopus and Google Scholar—to provide a broader perspective on the popularity of Beatles song titles in scientific literature, their development over the decades (starting from the early 1960s), and the relationship between total citations and the number of articles using Beatles song titles. Second, we expand our analysis by searching not only for exact song title matches but also for popular lyrics and instances of wordplay. To give an idea of the scope of our study: whereas previous research identifies approximately 550 relevant academic articles, our search in Scopus yields 2,048 articles containing exact Beatles song title matches and 555 that use song title wordplay. In addition, we find 189 article titles that contain an exact Beatles lyric and 408 that incorporate lyric-based wordplay. Beyond documenting this phenomenon, our study advances the understanding of how popular culture shapes academic communication (with implications for science communication, bibliometrics, and the science of science) and, as the first systematic cross-disciplinary analysis of wordplay in scientific article titles, demonstrates an LLM-based detection methodology that extends to other artists and cultural traditions.

Wordplay is central to this study, so we define it at the outset. Merriam-Webster defines *wordplay* as the “playful use of words: verbal wit” [12]. We adopt that sense and make it specific to our setting: a scientific article title is a wordplay on a Beatles song title or lyric when it playfully reworks that phrase while leaving the original recognizable. The rework may substitute, add, or drop a word, exploit a similarity in sound, or reinterpret the phrase in the technical context of the paper. ‘The lung and winding road’ [13], on platelet reactivity in Long COVID, turns ‘The Long and Winding Road’ on a single vowel; ‘Here comes the SU(N)’ [14] rereads ‘Here Comes the Sun’ as a mathematical group. A wordplay is not an *exact match*, in which the phrase is reused unchanged, and we report the two separately throughout. Nor is a title that merely shares words with a song, such as ‘Submarine groundwater discharge in the Yellow Sea’. We use the term broadly, covering puns as well as substitution, allusion, and reinterpretation.

Since this methodological approach sets our study apart from previous work, we will first discuss these studies in ‘Related work’. We outline our methodological choices in ‘Methods’. We then present the results of our analysis, in which we distinguish between four types of references: (1) exact song titles, (2) wordplay on song titles, (3) exact lyrics from Beatles songs (independent of the song title), and (4) wordplay on these lyrics. For each of these categories, we present the corresponding results in ‘Song title matches and wordplay’ and ‘Lyric matches and wordplay’. In the ‘Discussion’, we summarize our findings and reflect on their broader implications in science.

## Related work

In September 2014, it was revealed that a group of scientists at Sweden’s Karolinska Institute had been covertly inserting Bob Dylan lyrics into their research papers—part of a long-running bet among colleagues. The story first appeared in the institute’s internal magazine, KI-Bladet, but quickly gained global attention, making headlines in outlets such as The Washington Post and The Guardian [15]. The tradition began in 1997 with a review published in Nature Medicine, cleverly titled ‘Nitric oxide and inflammation: The answer is blowing in the wind.’ This revelation inspired Gornitzki et al. [1] to investigate whether other scientists had also embedded Dylan lyrics in biomedical literature. Using a comprehensive list of all Dylan’s songs and album titles, they searched for references in the titles of biomedical scientific publications indexed in the Medline database—the premier bibliographic resource for biomedical and life sciences literature. The two most frequently referenced Dylan songs identified were ‘The Times They Are A-Changin’ and ‘Blowin’ in the Wind’. The authors also examined truncated song titles of popular Dylan songs to uncover wordplay in scientific article titles. For example, ‘Knockin’ on pollen’s door: Live cell imaging of early polarization events in germinating Arabidopsis pollen,’ which is a pun on the 1973 Dylan song ‘Knockin’ on Heaven’s Door’. Inspired by [1], Brown et al. [16] published a small paper suggesting that meteorologists and climate impact scientists also reference Dylan lyrics. In a humorous twist, the authors embedded multiple Dylan song titles within the article’s text.

Rijkers et al. [11] extended this line of research by comparing the frequency of Dylan and Beatles song titles in biomedical literature. Their analysis found that Beatles references were more prevalent than Dylan’s, with ‘The Long and Winding Road’ being the most frequently referenced Beatles song and ‘The Times They Are A-Changin’ again leading among Dylan references. Notably, ‘The Long and Winding Road’ also appeared in non-biomedical fields, such as engineering and dentistry. Song titles consisting of a single word were excluded from the study, as the authors argued that it is difficult to establish a reliable connection between such titles and specific songs. To assess whether these musical references were intentional, the authors surveyed researchers whose publications included such titles. Approximately 50% of the respondents indicated that their aim was to make their paper stand out, while others noted that the lyrics of the title aptly matched the article’s content.

More recently, Nieuwenhuis [2] analyzed the frequent use of the Clash song ‘Should I Stay or Should I Go?’ in scientific publication titles. The author argued that the ‘publish or perish’ culture in academia may encourage researchers to choose creative or catchy titles—including popular song references—to attract attention. Furthermore, widely recognized cultural references are more likely to be rewarded than obscure ones, leading to repeated use of the same songs. By the time [2] appeared, 408 articles had referenced ‘Should I Stay or Should I Go?’, surpassing the references to the most popular Dylan (‘The Times They Are A-Changin’, 128 times) and Beatles (‘The Long and Winding Road’, 236 times) titles. However, we believe this comparison is flawed as the previous studies [1] and [11] focused exclusively on the biomedical domain.

Finally, Maci et al. [10] conducted a linguistic analysis of 546 biomedical article titles to explore the appeal of Beatles song references. The study evaluated the titles from three perspectives: (1) their level of informativeness; (2) the structure of information for meaning-making; and (3) the degree of accessibility and referential clarity. The authors found a clear preference for titles that creatively blend Beatles lyrics with biomedical themes, particularly when avoiding highly technical jargon. Their findings suggest that such titles act as attractors—attention-grabbing elements designed to create a memorable and engaging effect.

## Methods

We aim to identify academic research articles from any scientific discipline that contain references to the Beatles. Our analysis proceeds in two stages. First, we compile an overview of the number of articles whose titles include either the full title or a lyric from a Beatles song. Second, we examine article titles that constitute wordplay on a Beatles song title or lyric.

We begin with the comprehensive list of songs recorded by the Beatles, as compiled on Wikipedia [17]. Because deciding whether a given title intentionally references the Beatles is rarely clear-cut, we adopt a single guiding principle: a title is retained only when its appearance in an article title can be confidently attributed to the band rather than to ordinary language or coincidence. This criterion deliberately favors specificity over sensitivity—we accept that some genuine references are excluded in order to ensure that the retained titles are highly likely to be intentional.

We operationalize this principle through three criteria. First, we exclude all songs the Beatles did not themselves compose, even where their cover versions became widely recognized, such as ‘Twist and Shout’, ‘Roll Over Beethoven’, and ‘Long Tall Sally’, as an occurrence of such a title cannot be tied uniquely to the Beatles. Second, we exclude all single-word titles—such as ‘Yesterday’, ‘Wait’, ‘Girl’, ‘Rain’, ‘Birthday’, and ‘Because’—because an isolated common word can rarely be tied to a specific song without additional context (e.g., the opening lyric ‘All my troubles seemed so far away’ from ‘Yesterday’). Third, among multiple-word titles we retain only those whose wording constitutes an unambiguous reference to the Beatles (such as ‘With a Little Help from My Friends’), and we exclude titles that double as everyday English, whether fixed expressions or common phrases, since their appearance in an article title is at least as plausibly explained by ordinary usage as by deliberate allusion. Such excluded titles include ‘A Day in the Life’, ‘There’s a Place’, ‘All Together Now’, and ‘Run for Your Life’, all of which scored relatively high in an earlier version of our song titles list and data retrieval, with 1,149, 1,069, 260 and 35 hits, respectively. The Beatles may well have popularized expressions of this kind, but this does not imply that an author who uses one intends to invoke the band: ‘All Together Now’ can equally be read as a common English call for collective action, and it is unclear whether the frequent occurrence of ‘A Day in the Life’ reflects the track from ‘Sgt. Pepper’s Lonely Hearts Club Band’ or simply the idiom “a day in the life of…”. Where doubts arose, two of the authors independently classified all borderline titles, and disagreements were resolved by discussion. This criterion is stringent, but it is justified if the aim is to retain only titles that evoke a direct association with the Beatles in the reader’s mind, even though other authors have applied a less stringent test (see also [10]). S1 Table lists the resulting curated set of 112 Beatles songs.

An article title is considered an *exact match* if it contains the complete song title in the same word order. Minor variations are permitted, such as the omission of a definite or indefinite article (*the*, *a*, *an*) or a demonstrative adjective (e.g., *this*, *that*) at the beginning of the title. Additionally, conjugated forms of the verb *to be* (e.g., *is*, *are*) are treated as auxiliary and disregarded in the matching criteria, in alignment with how Scopus indexes content. For songs with a parenthetical subtitle, such as ‘Norwegian Wood (This Bird Has Flown)’, a match is accepted if either the main title ‘Norwegian Wood’ or the subtitle ‘This Bird Has Flown’ appears in the article title, unless the subtitle consists of generic words, in which case it is disregarded. For songs that include an initialism, such as ‘Back in the U.S.S.R.’, we also count matches where the periods are omitted (i.e., ‘Back in the USSR’). In fact, all matching is conducted case- and punctuation-insensitively. To reduce the likelihood of false positives, we only count matches in publications dated the same year or later than the release year of the corresponding song. This procedure is applied to both song titles and lyrics.

**Fig 1:**
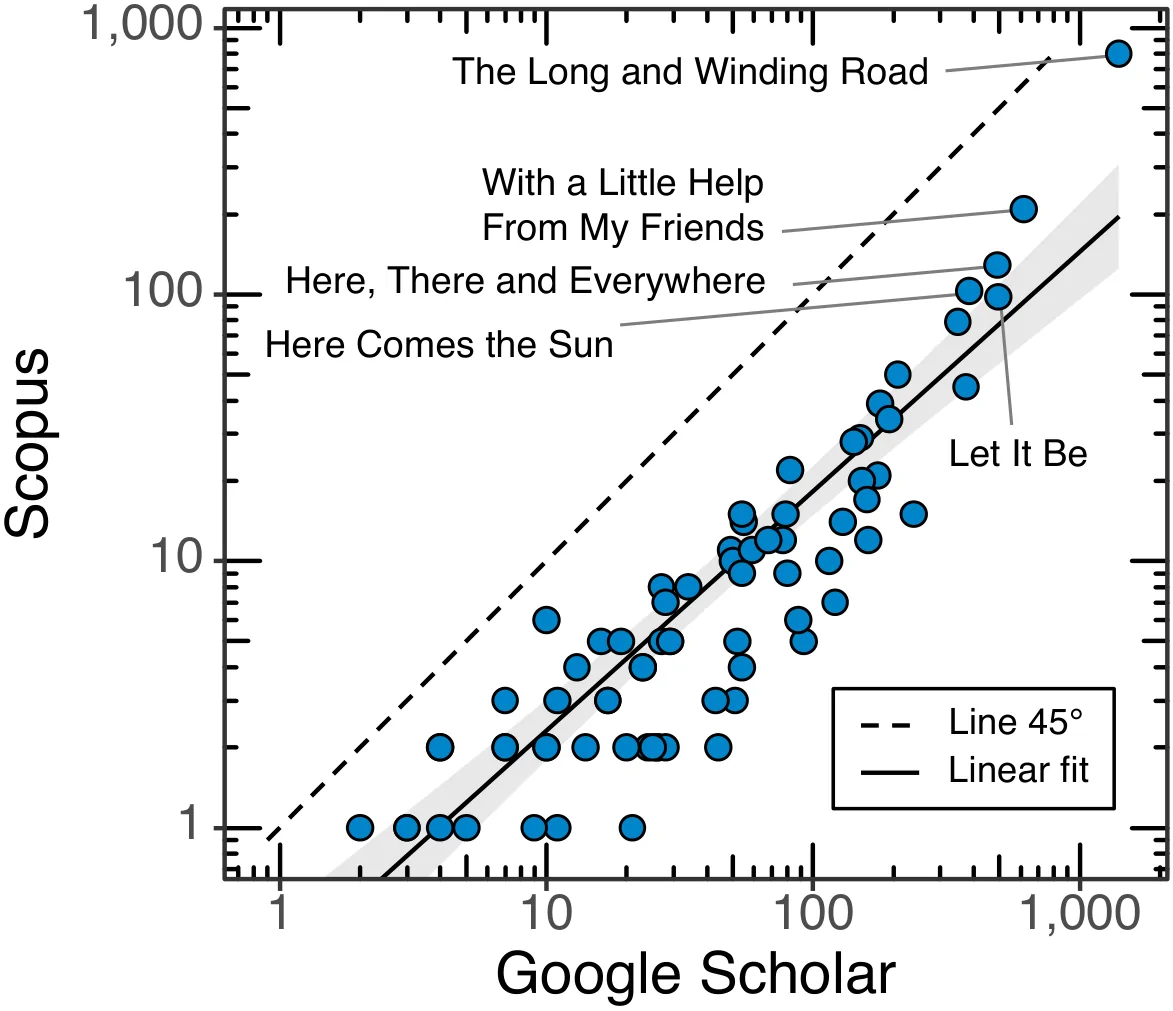
Comparison of exact matches of Beatles song titles in academic article titles on a log-log scale: Scopus vs. Google Scholar. The linear fit is drawn through the centers of 15 bins of equal logarithmic width (not shown) to minimize outlier impact; the shaded region represents the corresponding 95% confidence interval.

We retrieve academic publications from two databases: Scopus [18] and Google Scholar [19]. Scopus is a comprehensive abstract and citation database maintained by Elsevier, indexing only peer-reviewed scientific literature that meets specific editorial standards. It is widely used in bibliometric research and meta-analysis across disciplines. In contrast, Google Scholar is a freely accessible search engine that indexes scholarly content across a wide range of formats and publication types, including preprints and other non-peer-reviewed material. Unlike Scopus, Google Scholar does not support automated meta-research. The primary distinction between the two platforms is that Google Scholar includes unpublished or non-peer-reviewed work, such as preprints, while Scopus does not. We therefore base our analysis primarily on Scopus; as we show later, the reference counts obtained from the two databases are strongly correlated (Fig 1), confirming that Scopus provides a representative sample.

### Scopus

The main source for retrieving academic papers in this study is Scopus [18], accessed through the Scopus API [20]. We search for exact matches in academic article titles by leveraging the TITLE() function of the API and by enclosing the song title in double quotes. For ‘All You Need Is Love’, our search query is


$$\begin{array}{c} \text{TITLE("All You Need Is Love")}. \end{array}$$

For song titles with a parenthetical subtitle, we use the logical operator OR of the Scopus API. For ‘Norwegian Wood (This Bird Has Flown)’, our search query is


$$\begin{array}{c} \text{TITLE("Norwegian Wood") OR TITLE("This Bird Has Flown")}. \end{array}$$

Similarly, for song titles with an initialism, we also use the OR operator. For each query, we iterate over the search results and include only the articles with a publication year equal to or later than the release year of the song.

### Google Scholar

In our Google Scholar search queries, we use the advanced search functionality with the keyword allintitle to search for only exact matches in the titles of academic articles. For ‘All You Need Is Love’, our search query is


$$\begin{array}{c} \text{allintitle: "All You Need Is Love"}. \end{array}$$

We then filter the results by publication year using the as_ylo and as_yhi parameters in the URL. We record only the total number of retrieved results. For song titles containing a parenthetical subtitle or an initialism, we repeat the search using the same variations described in the ‘Scopus’ section, and then deduplicate and aggregate the resulting counts.

### Wordplay detection

We also retrieve academic articles whose titles contain a wordplay, in the sense defined in the ‘Introduction’, on Beatles song titles or lyrics. It is impossible to find such articles with the Scopus (or Google Scholar) search functionality directly. Large language models (LLMs) currently constitute the state of the art in semantic evaluation tasks [21]. Hence, we use an LLM to detect whether an academic article title is a wordplay on a Beatles song title.

As a starting point, we repeat the Scopus retrieval procedure described in the ‘Scopus’ section, but now relax the exact match requirement. We achieve this type of matching by executing the same queries but without the double quotation marks. These papers are all candidates for containing wordplay on Beatles song titles. To ensure data volume remains manageable, non-exact match results are capped at 1,000 papers per song title. Additionally, we repeat the exact-match search while omitting one word at a time, cycling through all words in the title. The search for wordplay candidates is constrained to single-word omissions within the Beatles lyrics.

We evaluate whether each of the resulting article titles is a wordplay on the corresponding song title or lyric with the gpt-4o-mini model through the OpenAI API. The temperature parameter of an LLM controls the randomness of the output. We set the temperature to 0.0, corresponding to no randomness, as is standard for classification tasks [22]. To optimize model performance, we employ a five-shot evaluation strategy following [23]. Specifically, the LLM is prompted with five human-annotated examples demonstrating the target task (in this case, identifying Beatles-related wordplay) before evaluating the test queries. In addition, we instruct the LLM to use Chain-of-Thought reasoning and provide an explanation before answering the question, which further improves the overall quality of the response [22]. We evaluate the LLM at the song level by passing all corresponding search results simultaneously. The LLM serves as a screening stage rather than the final arbiter: all titles it flags as wordplay are subsequently reviewed manually by the authors, and false positives are removed (false negatives encountered during this inspection are likewise corrected). The wordplay counts reported in this article therefore reflect human-verified instances only. We describe our approach and exact prompt in SI ‘Wordplay detection’. The complete pipeline, prompts, and resulting reference datasets are available in a GitHub repository [24]; the pipeline and prompts are also archived on Zenodo [25], and the resulting reference datasets on Zenodo [26].

### Validation of the wordplay classifier

To validate the wordplay classifier—that is, the raw LLM output before the manual verification described above—we drew a stratified random sample of 300 candidate titles [26]—150 that gpt-4o-mini labeled as wordplay and 150 that it labeled as not—and all three authors independently annotated each title as a wordplay on the corresponding Beatles song, or not, following a written guideline. Annotators saw only the song title and the article title; they worked blind to the model’s predictions and to one another’s labels, which rules out anchoring on the system being evaluated. Inter-annotator agreement was high (Fleiss’ κ=0.82, 95% CI [0.76, 0.88]; pairwise Cohen’s κ between 0.76 and 0.86), indicating that the construct is reliably identifiable. Taking the majority human label as ground truth, the classifier attains a recall of 0.83 and an *F*_1_ score of 0.59 (Table 1). Because the sample was balanced by the model’s own label, precision and the negative predictive value, which condition on the model’s prediction, are unbiased for the full candidate set: of the titles flagged as wordplay, 46% are confirmed by the human majority, and of those rejected, 91% are confirmed. Recall, accuracy, and *F*_1_, by contrast, depend on the true wordplay base rate and are conditional on the balanced sample.

**Table 1:** Validation of the wordplay classifier against human annotation (*n* = 300 titles).

Metric	Estimate (95% CI)
**Classifier vs. human majority**	
Precision	0.46 (0.38–0.54)
Negative predictive value	0.91 (0.86–0.95)
Recall (sensitivity)	0.83 (0.75–0.91)
Specificity	0.63 (0.56–0.69)
*F*_1_ score	0.59 (0.51–0.66)
Accuracy	0.68 (0.63–0.73)
Cohen’s κ	0.37 (0.27–0.46)
**Inter-annotator agreement** (all three authors)	
Cohen’s κ, RK–MT	0.85 (0.78–0.92)
Cohen’s κ, RK–GB	0.86 (0.79–0.92)
Cohen’s κ, MT–GB	0.76 (0.68–0.84)
Fleiss’ κ	0.82 (0.76–0.88)

Ground truth is the majority vote of the three authors, annotating independently and blind to the model’s predictions. Values in parentheses are 95% bootstrap confidence intervals. Precision and the negative predictive value condition on the model’s prediction and are unbiased for the full candidate set, whereas the remaining classifier metrics are conditional on the balanced (1:1) sampling design.

Inspecting the misclassifications, we find that false positives are dominated by misattributed wordplay: the title is a genuine pun, but on a different Beatles song, a different artist, or a non-musical source, rather than on the song supplied in the prompt. For instance, ‘Wake me up before you go-go… Can’t we do better with conscious sedation for endoscopy?’ was flagged for ‘You Can’t Do That’ but puns on Wham!’s ‘Wake Me Up Before You Go-Go’, and ‘Come now, let us treason together…’ was flagged for ‘All Together Now’ but alludes to the biblical ‘come now, let us reason together’. This indicates that gpt-4o-mini, as a comparatively small model, detects the presence of wordplay reliably but has limited capacity to condition strictly on the specific song and artist given in its context window. This is precisely the type of error that the manual verification stage removes: because every flagged title is reviewed by the authors, misattributed wordplay does not propagate to the counts reported in this article (see the ‘Discussion’ section).

## Song title matches and wordplay

### Scopus vs. Scholar

Fig 1 shows the number of exact matches of Beatles song titles in academic article titles on Scopus versus Google Scholar. The reference counts are plotted on a log-log scale to facilitate comparison across several orders of magnitude. The linear fit through the data points lies approximately parallel to the 45° line, indicating a strong correlation between the results obtained from Scopus and Google Scholar. Notably, all points fall below the 45° line, signifying that Google Scholar consistently yields higher reference counts than Scopus. This discrepancy is expected, as Google Scholar indexes a broader range of content, including non-peer-reviewed and unpublished work, whereas Scopus applies more stringent inclusion criteria, focusing solely on peer-reviewed literature. The Pearson correlation coefficient between the reference counts from the two databases is 0.93 when considering all songs, 95% CI [0.89, 0.95], *p* < 0.001; and 0.90 when computed on the log-transformed values of the non-zero counts, 95% CI [0.84, 0.93], *p* < 0.001. Given the strength of this correlation, we conclude that Scopus provides a sufficiently representative dataset and therefore use it exclusively for the remainder of the analysis.

### Most referred to song titles

Our final data collection was conducted on June 9, 2025. We believe that our list only contains titles where the authors unambiguously referred to Beatles songs due to our stringent selection criteria. The 20 most frequently referenced song titles are shown in Table 2. Interestingly, all four Beatles are represented in the list. Harrison scores highly with ‘Here Comes the Sun’, while Starr is indirectly represented as the originator of the phrase ‘A Hard Day’s Night’ (another Ringo-ism, ‘Tomorrow Never Knows’, did not chart in our list). The remaining results seem evenly split between Lennon and McCartney, although titles which can be associated with McCartney seem to have the edge over Lennon’s. This might seem a surprise, since Lennon was famous for his wordplay and even wrote two books to showcase this. However, ‘A Day in the Life’, a title strongly associated with Lennon, was removed from our list, as we explained. One conclusion we certainly cannot draw is that Lennon had less sensitivity to universally resonant expressions. On the contrary, we identified a substantial number of publications that echo variations of his ‘All You Need Is Love’, as will be further discussed in ‘Wordplay on song titles’. Given the scope of this article, our analysis was limited to Beatles songs. A follow-up study could investigate the cultural and academic impact of some of Lennon’s most iconic solo compositions. For example, a brief search in Scopus reveals a relatively high number of hits related to the signature Lennon phrase ‘Give Peace a Chance’ (31).

**Table 2:** The 20 most referred to Beatles song titles in academic article titles on Scopus.

Nr.	Song title	#	Examples
1	The Long and Winding Road	798	– Do black fintechs matter? The long and winding road to develop inclusive algorithms for social justice [36] – Vaccines for pseudomonas aeruginosa: A long and winding road [37]
2	With a Little Help from My Friends	209	– With a little help from my friends: Mitochondria maintain redox balance for the endoplasmic reticulum [38] – With a little help from my friends? Asymmetrical social influence on adolescent smoking initiation and cessation [39]
3	Here, There and Everywhere	129	– Here, there, and everywhere: Security analysis of Wi-Fi fine timing measurement [40] – Adenosine and inflammation: Here, there and everywhere [41]
4	Here Comes the Sun	103	– Here comes the sun: Physician relationships with industry and the Sunshine Act [42] – Here comes the sun—Is vitamin D a cure for all that ails us? [43]
5	Let It Be	98	– Let it be: Mindfulness and releasement—neglected dimensions of well-being [44] – Don’t let it be! Creative co-regulation and socially shared regulation in a case study of the Beatles: Get back [31]
6	Ticket to Ride	79	– They’ve got a ticket to ride: Xenorhabdus nematophila—Steinernema carpocapsae symbiosis [45] – A ticket to ride: The unintended consequences of school transport subsidies [46]
7	A Hard Day’s Night	50	– A hard day’s night: Time use in shift workers [47] – The British invasion phase: A hard day’s night [48]
8	All You Need Is Love	45	– All you need is love: Paul Ramsey’s basic Christian ethics and the dilemma of protestant antilegalism [49] – ‘All you need is love’ a social network approach to understanding attachment networks in adulthood [50]
9	Magical Mystery Tour	39	– Magical mystery tour: Salicylic acid signalling [51] – Fifty years of lipoprotein(a)—the magical mystery tour continues [52]
10	We Can Work It Out	34	– The continued advance of vaccine adjuvants—‘We can work it out’ [53] – We can work it out: Collaborative conflict resolution in model versioning [54]
11	Can’t Buy Me Love	29	– Can’t buy me love: Gift-giving among members of criminal organizations [55] – For money can’t buy me love? The political economy of marriages at the time of financialization [56]
12	Hello, Goodbye	28	– ‘Hello, goodbye’: Exploring the phenomenon of leaving teaching early [57] – Hello, goodbye: When do states withdraw from international organizations? [58]
13	Fixing a Hole	22	– Fixing a hole: A retrospective cohort study evaluating HAV, HBV, tetanus screening, and vaccination during hospitalization in persons who use substances [59] – Fixing a hole: Preventing pneumococcal pneumonia by vaccination [60]
14	Back in the U.S.S.R.	21	– Back in the USSR: Russian popular music in the times of military censorship [61] – Uzbek health care—no longer back in the USSR [62]
15	Drive My Car	20	– Product regulations: You can drive my car, otherwise let it be [63] – Robot, you can drive my car [64]
16	Nowhere Man	17	– “He is just the nowhere man of British politics”: Personal attacks in Prime Minister’s questions [65] – Nowhere man: The vain search for Harold Ford’s principles [66]
17	Glass Onion	15	– Looking through a glass onion: Exploring the validity of eye-tracking technology in capturing self-directed attention [67] – Glass Onion: Visual reasoning with recommendation systems through 3D mnemonic metaphors [68]
17	Norwegian Wood (This Bird Has Flown)	15	– Norwegian wood, isn’t it good? Narratives of the lumber industry and development paths in the Nordic periphery [69] – Slavic forest, Norwegian wood [70]
17	Strawberry Fields Forever	15	– Meat plants and strawberry fields forever? Precarious migrant labour in the German agri-food sector before and after COVID-19 [71] – Strawberry fields forever: The effect of farm-pollinator interaction network centrality on strawberry production [72]
20	Eight Days a Week	14	– Eight days a week: Long working hours, recovery, and breaks [73] – Eight days a week: The art of reference desk scheduling [74]
20	Yellow Submarine	14	– Are we living in a ‘Yellow Submarine’? The attitude and mood creation with the Beatles’ music [75] – We all need a yellow submarine! [76]

Regardless of whether they were consciously aware of it or not, the Beatles demonstrated an exceptional instinct for expressions with broad, universal appeal. Their optimistic tone, exemplified by songs like ‘We Can Work It Out’, ‘With a Little Help from My Friends’, and ‘Here Comes the Sun’, has likely contributed to this resonance. Ironically, there appears to be a tendency among scholars to invert that positive outlook, for example ‘Although it helps, love is not all you need: How Caryl Rusbult made me discover what relationships are all about’ [27], ‘Bacterial virulence: With a little help from my enemies’ [28], and ‘Can buy me love: The effect of child welfare expenditures on maltreatment outcomes’ [29]. Some authors also incorporate references to multiple Beatles songs within a single article title: ‘All together now, a magical mystery tour of the maize shoot’ [30] and ‘Don’t let it be! Creative co-regulation and socially shared regulation in a case study of the Beatles: Get back’ [31].

McCartney’s ‘The Long and Winding Road’ emerges as the winner among Beatles songs most frequently referenced in the titles of scientific articles. The total of 798 hits is striking. In our view, there is little doubt that these instances are deliberate references to the Beatles, even though we did encounter earlier uses of the phrase in online sources, predating 1970. Interestingly, the phrase *the long and winding road* also appears in the poem *The Return of Odysseus* by Scottish poet Edwin Muir, published in his *Collected Poems* (1952). It is known that Paul McCartney excelled in English during his school years, making it conceivable that he encountered the poem during his education and that the phrase remained with him. McCartney himself has remarked that the ‘winding road’ was an actual road leading to his Scottish farmhouse, and only later developed into a song. He stated that the phrase evokes “a double association of terrific sadness and also a sense of hope, particularly in the assertion that the road that ‘Leads to your door / Will never disappear”’ [32, p. 437]. Furthermore, according to its author, the song is ‘all about the unattainable; the door you never quite reach… the road that you never get to the end of’ [33, p. 539]. For scholars, it appears that this latter interpretation resonates most strongly. The metaphor of a *long and winding road* aptly captures the nature of the scientific process itself, which often progresses through trial and error toward meaningful, albeit temporary, results. Likewise, many of the phenomena studied in science are characterized by prolonged and unpredictable development.

The total of 798 references to the song clearly surpasses the 561 references (according to Scopus on July 27, 2025) to ‘Should I Stay or Should I Go?’, hence falsifying the claim of Nieuwenhuis [2] that this Clash song has more references than ‘The Long and Winding Road’. While our analysis was based exclusively on Scopus, a parallel exploration of Google Scholar reveals that entire research fields, such as breast cancer studies and cell differentiation, frequently cite this Beatles title in article headlines.

To substantiate our claim that the Beatles are the most frequently referenced musical artist in scientific article titles, we use Table 2 to compare them with other highly successful musical artists, as measured by record sales. For this we have used the list of best-selling music artists [34]. The top 3 of the list consists of our friends from Liverpool (claimed sales between 500 and 600 million), Michael Jackson (400–500 million) and Elvis Presley (500 million). For the comparison we have used the top 10 songs in Table 2, which amounts to 1,584 article titles referring to Beatles song titles. Next, we have looked at the top 50 most streamed songs on Spotify of both Michael Jackson and Elvis Presley, which are available through Kworb [35]. Each of these top 50 lists we curated in the same way as we curated the list of songs recorded by the Beatles, with the exception that we also allowed songs not written by the two artists themselves, as the majority of their songs were written by others. For both artists, we considered the top 10 of the resulting list, and determined all scientific articles referencing the given song titles, through Scopus, searching for publications up to June 9, 2025. For Michael Jackson, this yielded only 65 articles. The most referenced Michael Jackson song we found is ‘Man in the Mirror’. The same exercise for Elvis Presley led to 196 articles. The most referenced Elvis Presley song we found is ‘Suspicious Minds’. These numbers are far lower than 1,584, the number of articles we found referring to the top 10 Beatles songs in Table 2. This large difference persists despite focusing on their most popular songs and allowing broader inclusion criteria. The results highlight a markedly stronger presence of Beatles song titles in scientific literature than other top-selling artists.

### Distribution of Beatles references

**Fig 2:**
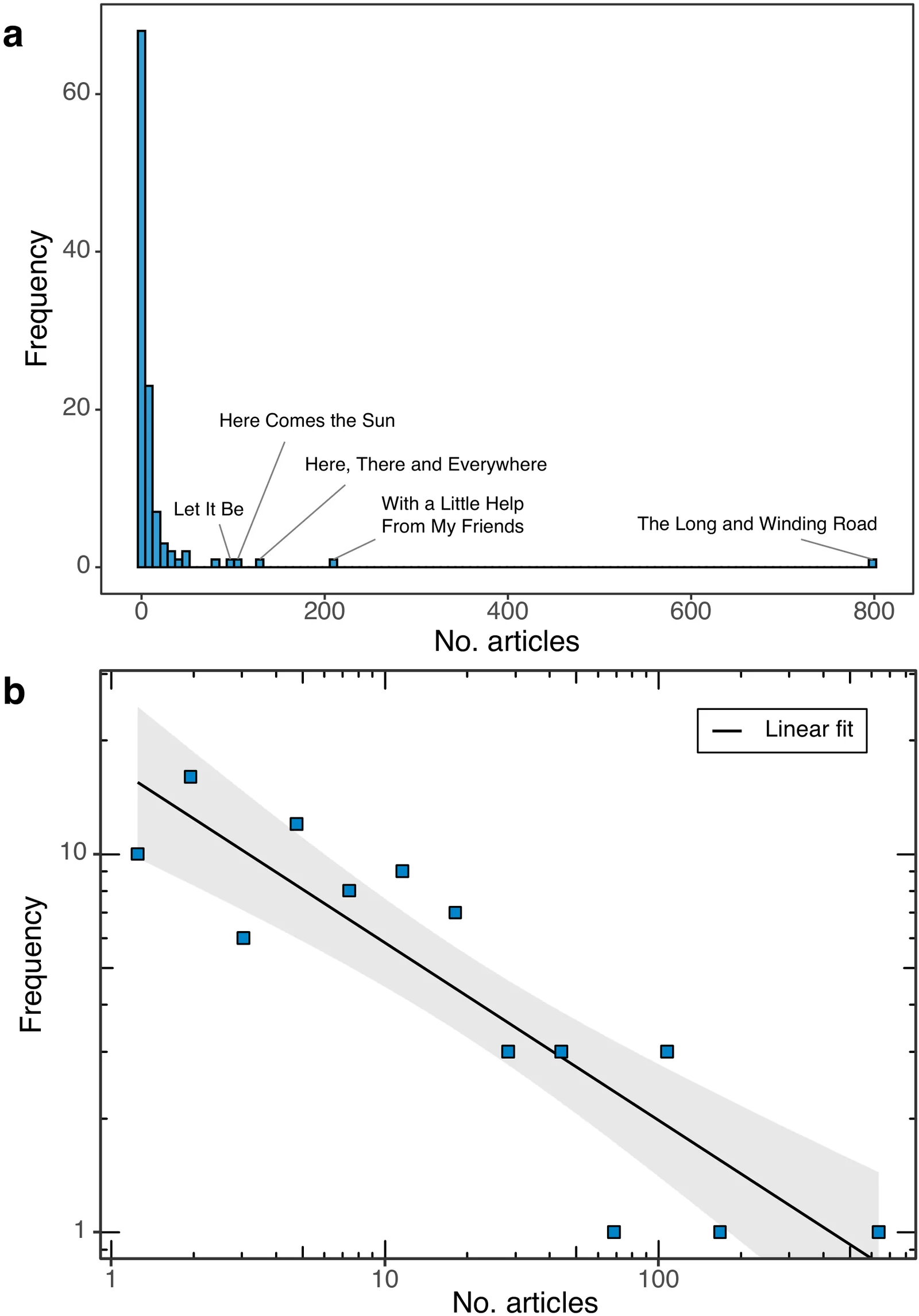
Distribution of Beatles song title references in academic article titles on Scopus. (a) The distribution of the number of papers per Beatles song title. (b) The same distribution shown with 15 bins of equal logarithmic width on a double logarithmic scale, where the bin centers fall approximately along a straight line (*R*^2^ = 0.795); the shaded area indicates the 95% confidence interval.

Retrieving the exact matches of Beatles song titles in Scopus yields 2,048 articles in total. Fig 2a shows the distribution of the number of papers per Beatles song title. The distribution is highly skewed: a few songs are referenced frequently, while most are only referenced a few times. Such a relationship is known as a power law or Zipf’s law. Power laws are found everywhere in nature, from the aftershocks of earthquakes to the structure of social networks and even the distribution of wealth—and now, it seems, in references to Beatles songs as well. Such power-law distributions appear as straight lines when plotted on a double logarithmic scale. Fig 2b shows the same distribution using 15 bins of equal logarithmic width on this scale, making the heavy-tailed nature of the data more clearly visible. The bin centers fall approximately along a straight line, *R*^2^ = 0.795.

### Disciplinary spread

**Fig 3:**
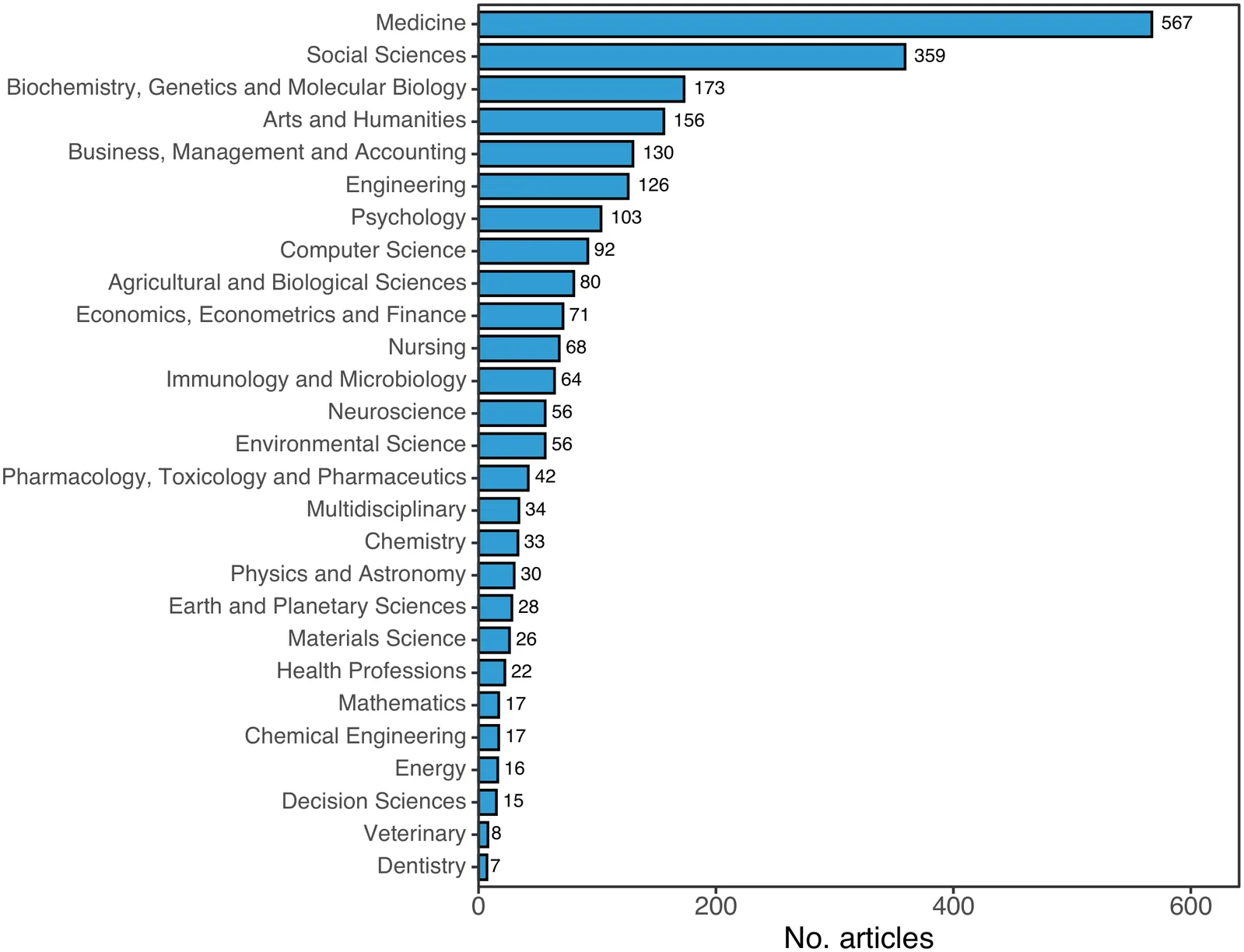
Subject areas of articles referencing the 10 most frequently matched Beatles song titles. Each article is assigned to one or more of the 27 Scopus subject areas, hence the bar counts sum to more than the number of articles.

The references are not confined to any single field. We classified the articles referencing the 10 most frequently matched song titles—which together account for 1,584 of the 2,048 exact song-title matches, or 77%—by their Scopus subject area. As Fig 3 shows, these articles span all 27 Scopus subject areas. Medicine and the Social Sciences dominate, but the long tail reaches fields as varied as Computer Science, Economics, Environmental Science, and the Arts and Humanities. This places the phenomenon well beyond the biomedical literature examined by earlier studies [10,11] and supports our characterization of it as cross-disciplinary.

### Beatles references per year

**Fig 4:**
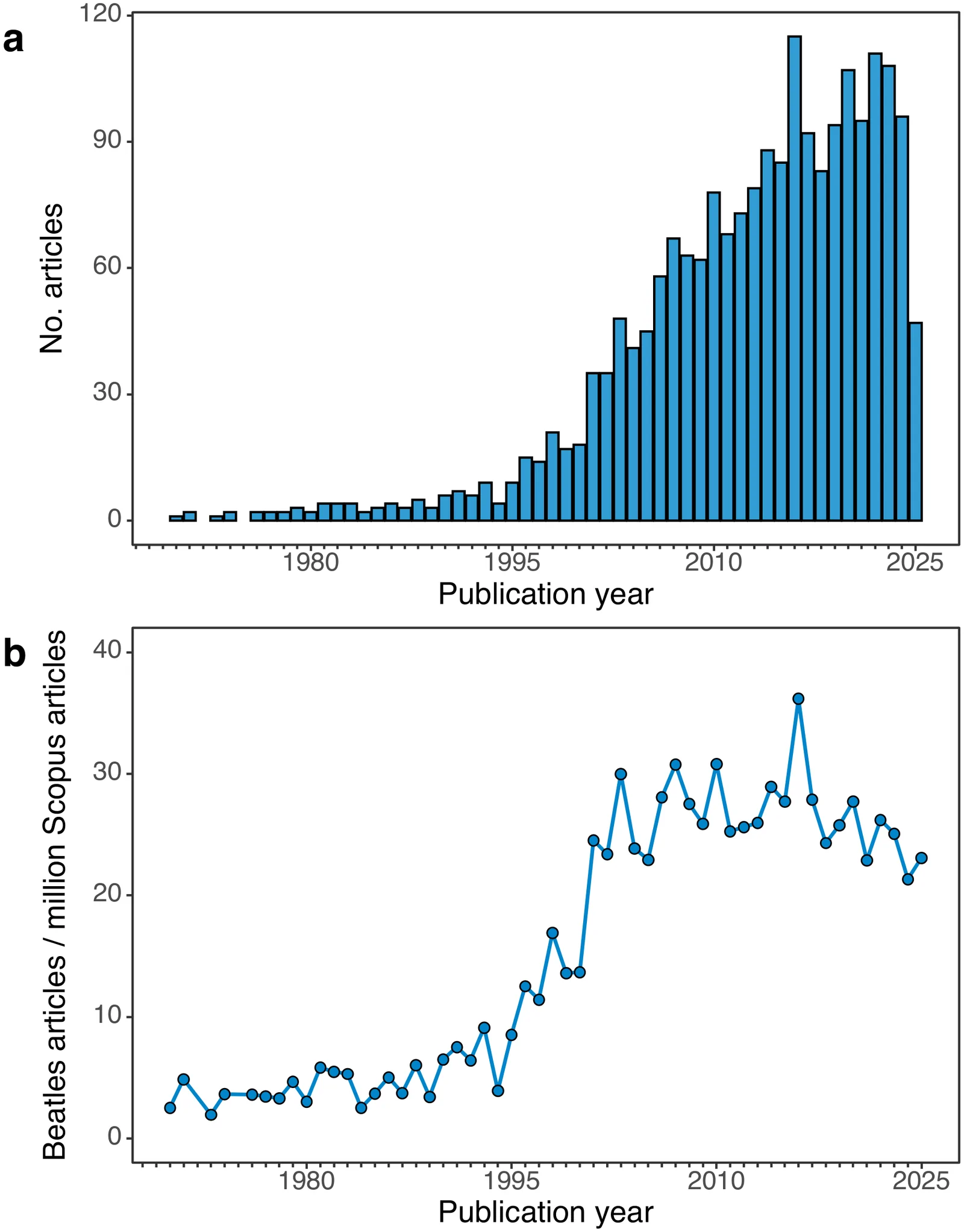
Academic articles per year referencing a Beatles song title. (a) Absolute number of academic articles per year with an exact reference to a Beatles song title in their titles. (b) The same counts normalized by the total number of articles indexed in Scopus each year (references per million articles), shown from the first nonzero year (1970) through 2025. Normalizing controls for the overall growth of the scientific literature: the Beatles’ share rises faster than the literature as a whole, increasing sharply between about 1995 and 2005 before leveling off. Note that the count for 2025 includes records through June 9, 2025.

Fig 4a tracks the rise of scientific articles featuring Beatles song titles over time. As with previous analyses, this graph is based on our curated list of song titles. The chart reveals only modest use of Beatles song titles up to around 1990. However, from the early 1990s onward, we observe a steady increase in the popularity of Beatles song titles in article titles.

A natural objection is that this increase might merely track the overall expansion of the scientific literature, which has grown enormously over the same period. To control for this, Fig 4b normalizes the annual counts by the total number of articles indexed in Scopus each year, expressing Beatles references as a relative frequency (references per million articles). The upward trend not only persists but becomes sharper: the Beatles’ share of article titles rose steeply between roughly 1995 and 2005 (increasing about threefold, from around 10 to 30 references per million) before leveling off. The growth in Beatles references therefore outpaced the growth of the scientific literature as a whole and cannot be explained by that expansion alone. This surge also coincides with the Beatles’ *Anthology* project (1995–1996) and the rapid digitalization of academic publishing, both discussed below. This naturally raises the question of how this trend can be explained.

We propose that the explanation lies in several independent but parallel developments that together reinforced the growing academic tendency to reference the Beatles.

The first development concerns the increasing popularity of the Beatles themselves. The death of John Lennon in 1980 marked the definitive end of any hope for a Beatles reunion. This event crystallized the Beatles era as a distinct cultural period that had lasted a single decade and would never return. That sense of historical closure prompted renewed attention to the Beatles across various domains. The stance of the former Beatles also contributed—consider George Harrison’s ‘When We Was Fab’ or Paul McCartney’s decision to fully embrace Beatles songs in his never-ending touring repertoire, a practice that had been more limited in the 1970s. The *Anthology* project (1995–1996) further boosted their popularity and introduced their music to new generations of listeners and musicians [77]. Many bands declared their artistic debt to the Beatles, sometimes even emulating their album artwork; for example, XTC’s *Oranges and Lemons* (1989). In the case of Oasis, the Beatles references are manifold, as can be heard in Richard Buskin’s podcast ‘Oasis v. The Beatles’, *Swinging through the sixties* (July 2018). The arrival of the Beatles on major streaming platforms like Spotify (in late 2015) has only reinforced this trend. It is thus plausible that academic research, like other sectors of society, began to reflect the Beatles’ cultural influence in the course of the 1990s.

We will not go further into the broader pop-cultural manifestations of the Beatles’ influence in the 1980s and 1990s. Our primary concern here is to account for the explosive rise in Beatles references in academic publications. This phenomenon, we argue, cannot be attributed solely to the band’s enduring popularity—nor, as the relative-frequency analysis above shows, to the general growth of the scientific literature. As noted at the beginning of this paper, the rise of the “publish or perish” culture is also relevant. While this imperative did not originate in the 1990s, the emerging digitalization of academic publishing during that decade greatly intensified it. In an increasingly crowded field, scholars faced growing pressure to stand out, and referencing one of the most globally recognized cultural phenomena became a natural and accessible rhetorical strategy. This trend coincided with a broader shift toward more informal language in scientific writing. The era in which academic papers were expected to be written exclusively in dry, impersonal prose is now firmly behind us. According to Hyland and others, this shift is part of the contemporary zeitgeist, whereby academic texts increasingly include personal commentary and colloquial elements [78].

The surge in Beatles references also has a clearly generational component. Fans of the 1960s and 1970s became academics themselves starting in the 1990s and eagerly drew on the soundtrack of their youth [11]. If not through article titles, then through catchy book covers—for example, the well-known series of molecular biology textbooks *Molecular Biology of the Cell* in which the authors recreated famous Beatles album covers, portraying themselves as John, Paul, George, and Ringo [79]. It seems likely that this tendency continued in subsequent generations: teenagers introduced to the Beatles through *Anthology* in the 1990s began referencing the band in their work in the 2010s. It remains to be seen whether the teenagers who first encountered the Beatles via streaming from 2015 onward will continue this upward trajectory.

Rijkers et al. [11], who conducted a survey on why authors in the biomedical sciences reference the Beatles, offer yet another explanation: “An increased interaction between popular culture and the biomedical scientific community.” There is little reason to believe that such interaction would be limited to biomedical research. Our bar chart shows that, since the 1990s, this phenomenon has extended across the academic spectrum and continues to grow. These references not only make texts more accessible and engaging but may also strengthen the connection between writer and reader. We hypothesize that references to well-known songs evoke a sense of shared cultural heritage and can encourage personal engagement between author and audience. One might argue that this effect is diminishing in the case of titles like ‘The Long and Winding Road’, a metaphor for a difficult research trajectory that has become something of a cliché. However, more inventive wordplay still offers appeal. For example, few fans of the *White Album* will fail to smile upon encountering titles such as ‘Happiness is a warm theorem’ [80] or ‘Everybody’s got something to hide except me and my patented monkey: Patentability of cloned organisms’ [81] in their professional literature.

### Citations

The 2,048 articles that contain an exact Beatles song title have collectively received 31,453 citations on Scopus. This number of citations represents the secondary scientific impact of the songs: it counts how often those articles have themselves been cited by later research.

**Fig 5:**
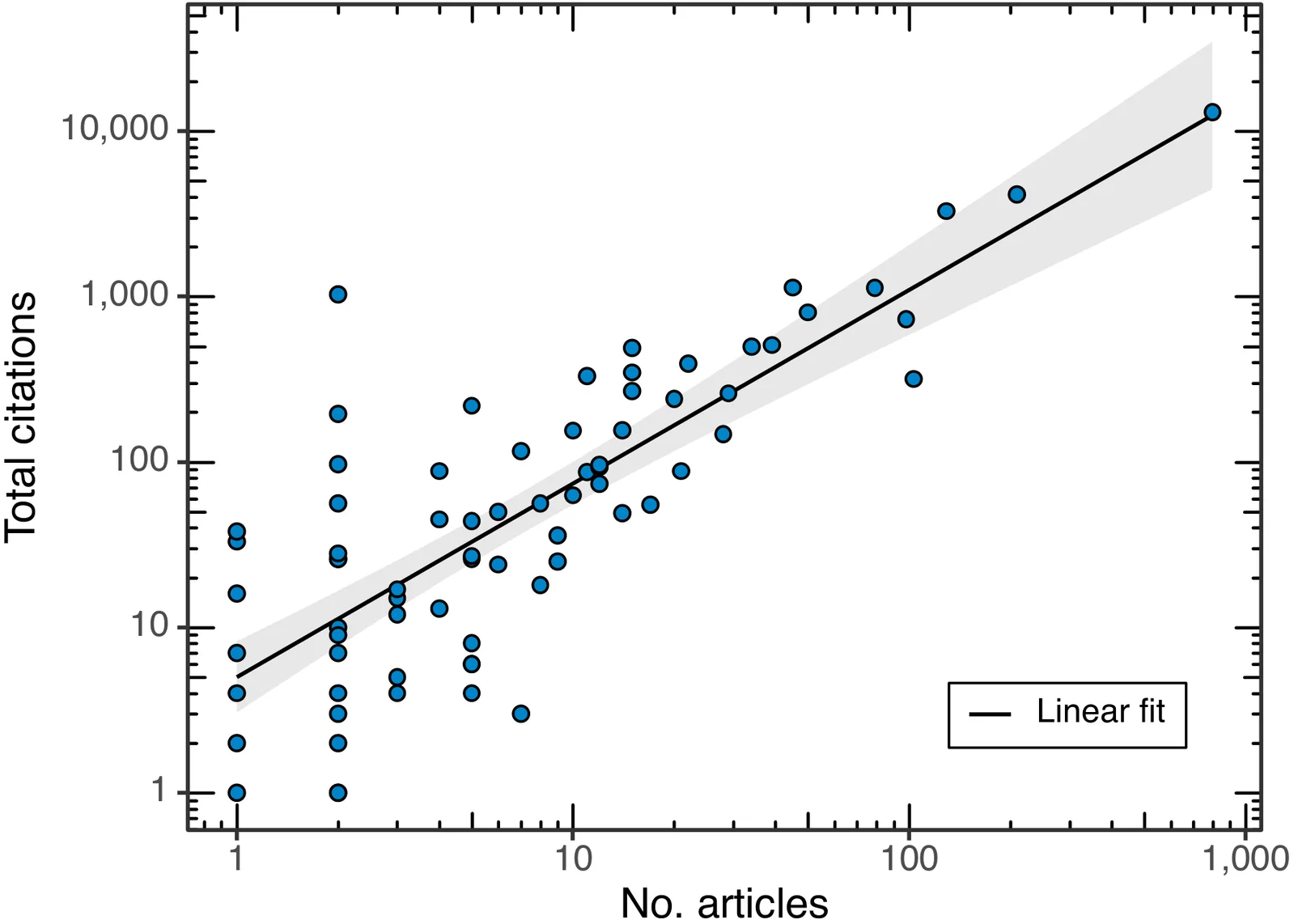
Total citations versus number of articles containing a Beatles song title. Shown on a log-log scale, where each point represents one song title, and the solid line shows the linear least-squares fit, with the shaded area indicating the 95% confidence interval.

Fig 5 depicts the connection between the number of articles in which a given song title appears (horizontal axis) and the cumulative citations those papers have accrued (vertical axis). On the doubly logarithmic scales, the points cluster around an upward-sloping line, *R*^2^ = 0.629, confirming a positive correlation. Titles occurring in more papers are, on average, cited more frequently. Aside from the expected larger scatter for song titles that are used less often, no systematic deviations from this trend are visible.

Particularly noteworthy are the three most highly cited articles in our dataset of exact matches, all published in reputable scientific journals. The article ‘Good day sunshine: Stock returns and the weather’ [82], which appeared in *The Journal of Finance*, illustrates the influence of sunny weather on stock returns while referencing a Beatles song in its title; it has been cited 1,009 times. ‘Seizure prediction: The long and winding road’ [83], published in *Brain* and cited 954 times, uses the song title to describe the challenges of seizure forecasting—the same usage pattern for ‘The Long and Winding Road’ as discussed in ‘Most referred to song titles’. Finally, ‘Th17 cell differentiation: The long and winding road’ [84], published in *Immunity* and cited 599 times, uses that same pattern again.

### Wordplay on song titles

Performing the Scopus retrieval procedure without the exact match requirement, by repeating each query while leaving out one title word at a time, and then excluding the exact matches, yields a total of 20,058 articles. Since we no longer enforce exact matches in the Scopus queries, an article title may now appear in the search results for multiple song titles, producing a large pool of candidates for wordplay. Because most of these hits are merely coincidental matches, the titles must be chosen with particular care.

After applying wordplay tagging with the LLM, manually checking for false positives and negatives, and deduplicating the results, 555 unique article titles were found to contain Beatles song-title wordplay. Table 3 shows the Beatles song titles most frequently referenced through wordplay. We observe the same pattern as in the exact matches: a handful of song titles lend themselves especially well to wordplay. Some article titles identified as wordplay are merely alternative spellings of a song title, such as the phrase ‘when I’m 64’ as a match for ‘When I’m Sixty-Four’. As these article titles did not qualify for an exact match, we included them here anyway. The full list of 555 articles with wordplay is available in the accompanying data repository [26].

**Table 3:** Beatles song titles most frequently referenced through wordplay in academic article titles.

Nr.	Song title	#	Examples
1	All You Need Is Love	137	– All you need is sleep [85] – Targeting sirtuin 1 to improve metabolism: All you need is NAD+? [86]
2	With a Little Help From My Friends	91	– With(out) a little help from my friends? Reconciling incongruous findings on stakeholder management, innovation, and firm performance [93] – Bacterial virulence: With a little help from my enemies [28]
3	The Long and Winding Road	76	– Myocyte hypertrophy: The long and winding RhoA’d [87] – The lung and winding road: Inflammation-driven platelet reactivity in Long COVID [13]
4	Here, There and Everywhere	59	– Hair, there, and everywhere: Peritonsillar abscess formation initiated by pet hair ingestion [94] – Here, there, and in-between: The poetic narratives of a transnational family’s translingual, multiliterate, and trans-geographic reality [95]
5	When I’m Sixty-Four	46	– Will you still need me, will you still screen me, when I’m past 64? Breast screening policy is based on ageism [96] – When I’m 84: What should life look like in old age? [97]
6	Can’t Buy Me Love	25	– Money can’t buy me hygge: Danish middle-class consumption, egalitarianism, and the sanctity of inner space [98] – ‘Money can’t buy me land’: Foreign land ownership regime and public opinion in a transition society [99]
7	Here Comes the Sun	21	– Here comes the SU(N): Multivariate quantum gates and gradients [14] – Here comes the son: A Shandean project [100]
8	Let It Be	18	– Let it “B”? The role of Hepatitis B universal vaccination among Italian problematic drug users [90] – Let it bee: A case study of applying triadic game design for designing virtual reality training games for beekeepers [101]
9	Hello, Goodbye	14	– Goodbye Dr. Google, hello Dr. ChatGPT: Advancing a new frontier for ENT medical information online [102] – Goodbye substantial factor, hello Doull v. Foster! [103]
10	A Hard Day’s Night	11	– A hard day’s work: Measuring women’s work in anthropological research [104] – A hard day’s write: Beatles fanfic and the quantum of creativity [105]
10	Magical Mystery Tour	11	– The concept of *Sebestoimost’* in Russian farm accounting: A very unmagical mystery tour [106] – A mathematical mystery tour: Higher-thinking math tasks (grades 5–12) [107]
12	Lucy in the Sky with Diamonds	10	– Lucy in the sky without diamonds: Stealing confidential data in the cloud [88] – Leucine in the sky with diamonds [89]
13	Strawberry Fields Forever	8	– Chrysanthemum fields forever: The labor exchange band, Taiwanese folk rock, and the LP form [108] – Hot spots, avatars, and narrative fields forever: Bunuel’s legacy for new digital media and interactive database narrative [109]
14	Happiness Is a Warm Gun	7	– Happiness is a warm… customer [110] – Happiness is a warm theorem [80]
15	Ticket to Ride	5	– Ticket to a smoother ride [111] – Ticket to divide: m^6^A reader YTHDC1 drives acute myeloid leukemia proliferation [112]
16	Everybody’s Got Something to Hide Except Me and My Monkey	2	– Everybody’s got something to hide except me and my patented monkey: Patentability of cloned organisms [81] – Everybody’s got something to hide except me and my NFT monkey: Analysis of the european commission’s DAC8 proposal on automatic exchange of crypto-asset information [113]
16	While My Guitar Gently Weeps	2	– While my guitar gently reaps [114] – Still my guitar gently weeps. Questions for an ockhamized metaphysics of the event sources of sound [115]
16	Love Me Do	2	– Bind Me Tender, Bind Me Do!: Dative and accusative arguments as antecedents for reflexives in Polish [116] – From experiencer verbs to Agree-and-Move: A review of Bind Me Tender, Bind Me Do! [117]
19	Do You Want to Know a Secret	1	– Do you want to know a trade secret—How Article 2B will make licensing trade secrets easier (but innovation more difficult) [91]
19	Revolution 9	1	– Rated X: Apple’s new operating system aims to score a perfect 10, but for some, Revolution No. 9 was change enough [118]
19	I’ll Follow the Sun	1	– I’ll follow the fun: The extended investment model of social media influencers [92]
19	Norwegian Wood (This Bird Has Flown)	1	– Bird flu has flown [119]
19	I Me Mine	1	– I, me, me, mine!: Autobiographical fiction and the “I” [120]
19	I’ve Just Seen a Face	1	– ‘I have just seen a face of old Africa’: The depiction of black Africa in National Geographic magazine [121]
19	Maxwell’s Silver Hammer	1	– Grenville’s silver hammer: The problem of money in the Stamp Act crisis [122]
19	Nowhere Man	1	– The nowhere (wo)man: An example of the defensive use of emptiness in a patient with a schizoid disorder of the self [123]
19	Please Please Me	1	– Please, please, just tell me: The linguistic features of humorous deception [124]
19	We Can Work It Out	1	– Can Wii work it out? [125]

Many variations on ‘All You Need Is Love’ made their way into the academic literature, such as ‘All you need is sleep’ [85] and ‘Targeting sirtuin 1 to improve metabolism: All you need is NAD+?’ [86]. Similarly, humorous wordplays on ‘The Long and Winding Road’ are found in ‘The long and winding RhoA’d’ [87] and ‘The lung and winding road: Inflammation-driven platelet reactivity in Long COVID’ [13]. Sometimes wordplay is achieved by simply interpreting a song title creatively, such as in ‘Here comes the SU(N): Multivariate quantum gates and gradients’ [14], where the authors have linked SU(N), a mathematical framework from quantum computing, to a similar-sounding word in the Beatles song title. Songs with fewer wordplay article titles often require creativity, such as in ‘Lucy in the sky without diamonds: Stealing confidential data in the cloud’ [88] or ‘Leucine in the sky with diamonds’ [89], which are clever plays on the song title ‘Lucy in the Sky with Diamonds’. Among the more striking examples are ‘With a little help from my enemies’ [28], ‘Let it “B”? The role of Hepatitis B universal vaccination among Italian problematic drug users’ [90], ‘Happiness is a warm theorem’ [80], ‘Do you want to know a trade secret’ [91], and ‘I’ll follow the fun’ [92].

The list of wordplay titles in Table 3 is probably incomplete, since our search captures only those titles that either (i) contain every non-auxiliary word from the original song but in a different order, or (ii) contain all but one word in the same order.

## Lyric matches and wordplay

### Most referred to lyrics

Our approach to compiling a list of Beatles lyrics was less structured than the one used for song titles. We began by selecting approximately 20 characteristic and widely recognized phrases from Beatles lyrics, such as ‘I read the news today, oh boy’ (‘A Day in the Life’), ‘Speaking words of wisdom’ (‘Let It Be’), and ‘There are places I remember’ (‘In My Life’). We then expanded this list to 34 phrases by consulting ChatGPT for additional well-known lyrics. Using this final list, given in S2 Table, we applied the same Scopus retrieval procedure, enforcing an exact matching criterion. The results, summarized in Table 4, show a total of 189 matches. The final data collection was completed on July 6, 2025.

**Table 4:** Beatles lyrics most frequently referenced in academic article titles.

Nr.	Lyric (song)	#	Examples
1	You say you want a revolution *Revolution*	53	– The time is now for gene- and genome-based bacterial diagnostics: ‘You say you want a revolution’ [131] – You say you want a revolution: Two notions of probabilistic independence [127]
2	Come together, right now *Come Together*	23	– Come together, right now! ZCCHC3 orchestrates cytosolic nucleic acid sensing through phase condensation [132] – Come together, right now: Retweeting in the social model of protest mobilization [133]
3	It’s getting better all the time *Getting Better*	21	– It’s getting better all the time? Using secular trends to understand the impact of neurocritical care [134] – The hyphen as a segmentation cue in triconstituent compound processing: It’s getting better all the time [135]
4	Help! I need somebody *Help!*	15	– Help, I need somebody: Examining the antecedents of social support seeking among cybercrime victims [136] – Help! I need somebody… Help! Not just anybody: Applying recommender system techniques in peer help systems [137]
5	Love is all you need *All You Need Is Love*	13	– The new backlash: Popular culture’s ‘marriage’ with feminism, or love is all you need [138] – Love is all you need: User preferences of strategies for mediating intimate relationships through technology [139]
6	Try to see it my way *We Can Work It Out*	11	– Try to see it my way: Humans take the level-1 visual perspective of humanoid robot avatars [128] – Try to see it my way: Embodied perspective enhances self and friend-biases in perceptual matching [140]
6	Will you still need me, will you still feed me *When I’m Sixty-Four*	11	– Microbial mutualism: Will you still need me, will you still feed me? [130] – Gerontology: Will you still need me, will you still feed me? [141]
8	I read the news today, oh boy *A Day in the Life*	9	– I read the news today, oh boy: The effect of crime news coverage on crime perception [129] – ‘I read the news today… oh boy’ Taking the pulse of UK popular music journalism [142]
9	Something in the way she moves *Something*	7	– ‘Something in the way she moves’: The functional significance of flexibility in the multiple roles of protein disulfide isomerase (PDI) [143] – Something in the way she moves: Biological motion, body shape, and attractiveness in women [144]
10	Living is easy with eyes closed *Strawberry Fields Forever*	4	– ‘Living is easy with eyes closed…’ on blinded RCTs and specific and non-specific effects of complex therapeutic interventions [145]
10	Get back to where you once belonged *Get Back*	4	– ‘Get back to where you once belonged’? Effects of skilled internal migration on Italian regional green growth [146]
12	Take a sad song and make it better *Hey Jude*	3	– Take a sad song and make it better: Spousal activity limitations, caregiving, and depressive symptoms among couples [147]
12	You say yes, I say no *Hello, Goodbye*	3	– You say yes, I say no: Investigating the link between meaning and form in response particles [148]
14	When I find myself in times of trouble *Let It Be*	2	– ‘When I find myself in times of trouble…’: The conditional effect of international organisations on policy convergence [149]
14	I’d like to be under the sea *Octopus’s Garden*	2	– ‘I’d like to be under the sea’: Contextual cues in virtual environments influence the orientation of idea generation [150]
14	Look at all the lonely people *Eleanor Rigby*	2	– ‘Ah, look at all the lonely people…’: Loneliness and solitude in public mental health [151]
14	Nothing’s gonna change my world *Across the Universe*	2	– Nothing’s gonna change my world—Or do journalistic clarifications help against rumors? [152]
14	Speaking words of wisdom *Let It Be*	2	– Speaking words of WISDOM: Web Intelligent Search based on DOMain ontologies [153]
19	Let me tell you how it will be *Taxman*	1	– Let me tell you how it will be; Here’s one for you, nineteen for me: Modifying the internal revenue service’s approach to resolving tax disputes [154]
19	Let me take you down *Strawberry Fields Forever*	1	– Let me take you down [155]

The first thing that stands out is that several song titles we previously omitted because they didn’t meet our strict selection criteria do appear in Table 4. Examples include ‘Help! I need somebody’ and ‘Come together, right now’. The number of articles with ‘You say you want a revolution’ in the title—namely, 53—would even be high enough to earn a place in the top 10 of Table 2. Still, the number of matches reported in Table 4 is much smaller than that in Table 2. This is in line with [126], which states that the semantic memory for song titles is better than for lyrics. We also note that the majority of the lyrics in Table 4 are opening lines from Beatles songs. Quite often, these opening lines are also the opening lines of the article titles, for example ‘You say you want a revolution: Two notions of probabilistic independence’ [127], ‘Try to see it my way: Humans take the level-1 visual perspective of humanoid robot avatars’ [128] and ‘I read the news today, oh boy: The effect of crime news coverage on crime perception’ [129]. Another observation from Table 4 is that quite some lyrics are from choruses of Beatles songs, for instance ‘Microbial mutualism: Will you still need me, will you still feed me?’ [130] (containing a line from the chorus of ‘When I’m Sixty-Four’). The repetitive and simple nature of choruses may help explain why their lyrics tend to be more memorable than those found in verses. Among the lyrics for which we did not find any article titles are ‘I think I’m gonna be sad’ (‘Ticket to Ride’) and ‘There are places I remember’ (‘In My Life’). Because our search for article titles referencing Beatles lyrics was less structured, we expect that additional examples could be readily found.

### Wordplay on lyrics

Table 5 lists the 408 article titles we identify as wordplays on Beatles lyrics. We observe that the lyric ‘Love is all you need’ appears in numerous wordplays of a specific format, for which we provide an explanation below. Besides, we note some creative wordplays, like ‘Help! I need WNT antibody. Help! Not just any antibody’ [156], a wordplay on ‘Help! I need somebody’ and the subsequent line ‘Help! Not just anybody’, or ‘Fieldwork is easy with eyes closed, misunderstanding all you see…’ [157], a wordplay on ‘Living is easy with eyes closed, misunderstanding all you see’. Some article titles listed in Table 5 are merely alternative formulations of a lyric, like ‘words of wisdom’ and ‘getting better all the time’. As these article titles did not qualify for an exact match, we included them here anyway.

**Table 5:** Beatles lyrics most frequently referenced through wordplay in academic article titles.

Nr.	Lyric (song)	#	Examples
1	Love is all you need *All You Need Is Love*	305	– Attention is all you need [159] – RNA is all you need [158]
2	It’s getting better all the time *Getting Better*	49	– Machine learning in mental health—getting better all the time [160] – ${\text{FFR}}_{\text{CT}}$: Getting better all the time (but not there yet) [161]
3	Something in the way she moves *Something*	14	– Something in the way she sings: Enhanced memory for vocal melodies [162] – Something in the way we move: Motion dynamics, not perceived sex, influence head movements in conversation [163]
3	You say you want a revolution *Revolution*	14	– You say you had a revolution: Methodological foundations of closed-loop psychology [164] – You say you want a devolution? Lessons from the Main Street Program [165]
5	Help! I need somebody *Help!*	13	– HELLP syndrome: ‘I need somebody HELLP, not just anybody…' [166] – Help! I need a WNT antibody. Help! Not just any antibody [156]
6	Will you still need me, will you still feed me *When I’m Sixty-Four*	4	– Will you still need me, will you still read me…? Patron-driven acquisition books’ circulation advantage long-term and post-pilot [167] – The aging workplace—It’s not just ergonomics or will you still need me, will you still love me, when I’m 64? [168]
7	Speaking words of wisdom *Let It Be*	3	– John Launer: Hearing words of wisdom [169] – Speaking words of life [170]
8	Living is easy with eyes closed *Strawberry Fields Forever*	2	– Fieldwork is easy with eyes closed, misunderstanding all you see… and other reflections: 3ie annual Howard White inaugural lecture [157] – Is living easier with eyes closed? [171]
9	You say yes, I say no *Hello, Goodbye*	1	– You say ‘PK’ and I say ‘no way!’; You say ‘keo’ and I say ‘time to go!’ [172]
9	I read the news today, oh boy *A Day in the Life*	1	– ‘I shared the news today, oh boy’: News provision and interaction on Facebook [173]
9	Look at all the lonely people *Eleanor Rigby*	1	– Look (closely) at all the lonely people: Age and the social psychology of social support [174]
9	We all live in a yellow submarine *Yellow Submarine*	1	– We all live in a virtual submarine [175]

For the lyric ‘Love is all you need’, we observe a very specific pattern in the wordplays after 2017, even though the pattern also appeared before that, e.g., ‘RNA is all you need’ [158]. The 2017 article ‘Attention is all you need’ by Vaswani et al. [159] was cited 89,737 times on Scopus—nearly three times the total number of citations received collectively by the 2,048 articles featuring a Beatles song title in their titles, as listed in the ‘Citations’ section. According to Google Scholar, the article [159] was cited 184,801 times, an unprecedented number for a scientific publication. Vaswani et al. introduced the Transformer-based language model architecture featuring a mechanism called *self-attention*, which laid the foundation for large language models such as ChatGPT and catalyzed the subsequent AI boom in both research and industry [21]. The article ‘Attention is all you need’ is one of the most influential scientific publications of the 21^st^ century to date. From the 305 articles that are a wordplay on ‘Love is all you need’, all but 14 articles appeared after 2017. As most of them are in the field of computer science, it is more likely that they are wordplays on ‘Attention is all you need’, which is itself, in turn, a wordplay on the Beatles lyric ‘Love is all you need’.

## Discussion

The Beatles are present in the scientific literature to a degree that we found surprising. Using a curated set of their songs and lyrics, we identified more than 2,200 exact references and over 900 instances of wordplay in article titles spanning many disciplines. To our knowledge, this is the first cross-disciplinary account of how the band’s music and words have spread through scientific writing. This prominence is not merely a byproduct of musical fame: the top 10 Beatles song titles appear in 1,584 article titles, an order of magnitude more than the equivalent top 10s of Michael Jackson (65 articles) and Elvis Presley (196), the two next best-selling artists. These references reflect both the Beatles’ lasting popularity and a change in how scholarship is written, as authors increasingly draw on popular culture and wordplay to make their titles memorable in a crowded field.

The most frequently referenced song titles—‘The Long and Winding Road’, ‘With a Little Help from My Friends’, and ‘Here, There and Everywhere’—are themselves apt metaphors for the scientific process: for the course of research, for collaboration, and for discovery. The use of Beatles references gained momentum from the 1990s onward, with several plausible drivers: a generation raised on the band entering academia, a broader move toward informal academic writing, and the “publish or perish” incentive to coin memorable titles. This rise holds even after we normalize for the overall growth of scientific output: the Beatles’ share of article titles climbed sharply between roughly 1995 and 2005 and has since plateaued. In other words, referencing the Beatles has become an established feature of academic writing rather than one that keeps accelerating.

Wordplay and references to well-known lyrics (‘Love is all you need’, ‘Speaking words of wisdom’, and ‘You say you want a revolution’) show that this reach extends beyond song titles. In some cases these lyrics have even lent their phrasing to the titles of landmark publications, as with the 2017 article ‘Attention is all you need’ by Vaswani et al. [159], cited nearly 90,000 times on Scopus. That paper, among the most cited of the 21^st^ century to date, laid the groundwork for large language models such as ChatGPT and helped set off the subsequent AI boom in research and industry [21].

Our study naturally has some limitations. First, our search is restricted to publication titles indexed in Scopus and, therefore, inherits its coverage and indexing conventions. Second, the lyric analysis is necessarily non-exhaustive (we searched a curated set of phrases rather than all possible lyrics, and our wordplay heuristics capture only certain structural variations), so the true number of references is likely higher than what we report. Third, our strict matching and exclusion criteria prioritize specificity over sensitivity: by requiring clear, unambiguous evidence of intentional references and wordplay, we inevitably exclude many genuine matches, particularly those involving short or common phrases, in order to minimize false positives. Finally, although we validated the automated wordplay detection against independent, blinded human annotation (Table 1), it remains imperfect, in part because we relied on a comparatively small language model (gpt-4o-mini). Its false positives are dominated by misattributed wordplay—genuine puns, but on a different Beatles song, a different artist, or a non-musical source rather than the song we supplied—which indicates that the model detects the presence of wordplay reliably but struggles to attribute it to the specific song and artist. Because all titles flagged by the model were subsequently verified by hand and false positives removed, such misattributions do not enter the reported counts; the residual error lies instead with genuine wordplay that the model failed to flag, so our wordplay counts are, if anything, conservative. In addition, our citation figures are descriptive and do not establish that Beatles references cause higher citation counts.

These patterns also carry practical lessons. The clearest is for authors: a well-chosen cultural reference can make a technical contribution more memorable, as long as the allusion fits the content without sacrificing clarity for readers or discoverability in literature searches. The same titles can help editors and science communicators reach past a specialist audience, since a shared cultural touchstone travels easily between fields. And for those who study scholarly communication and bibliometrics, the cross-disciplinary spread we document is concrete evidence that academic writing is becoming more informal and more attentive to its readers.

Decades after the band’s heyday, the Beatles still find their way into the titles of scientific papers. When researchers reach for a familiar song, they borrow a phrase their readers already know and, in doing so, reach out to them. How far such references actually shape whether a paper is found, read, and remembered is a question worth pursuing. But our results already suggest that, now and then, science really does get by with a little help from the Beatles.

## Supporting information

S1 Table Included songs by the Beatles in alphabetical order. The curated set of 112 Beatles songs used for the song-title analysis, with the release year of each song.
(PDF)

S2 Table Selected Beatles lyrics and their source song. The 34 Beatles lyric phrases used for the lyric analysis, each paired with the song from which it is taken.
(PDF)

S1 Appendix Wordplay detection. Details of the large language model used to detect wordplay, including the generic system and user prompt templates and a worked example of a user prompt with the corresponding model output.
(PDF)
