# Supplementary material for: How science gets by with a little help from the Beatles: Detecting cultural wordplay in scientific literature using large language models
Source: PLoS One. 2026 Sep 9;21(9):e0356147. doi: 10.1371/journal.pone.0356147 (PMC13557405; doi:10.1371/journal.pone.0356147)
Supplement: Supplementary file 1 [file pone.0356147.s001.pdf]

**S1 Table. Included songs by the Beatles in alphabetical order.**

| A–I | | I–Z | |
|---|---|---|---|
| **Song** | **Year** | **Song** | **Year** |
| All My Loving | 1963 | I'm So Tired | 1968 |
| All You Need Is Love | 1967 | I've Just Seen a Face | 1965 |
| And I Love Her | 1964 | Komm, Gib Mir Deine Hand | 1964 |
| And Your Bird Can Sing | 1966 | Lady Madonna | 1968 |
| Baby, You're a Rich Man | 1967 | Let It Be | 1970 |
| Baby's in Black | 1964 | The Long and Winding Road | 1970 |
| Back in the U.S.S.R. | 1968 | Love Me Do | 1962 |
| The Ballad of John and Yoko | 1969 | Lovely Rita | 1967 |
| Being for the Benefit of Mr. Kite! | 1967 | Lucy in the Sky with Diamonds | 1967 |
| Blue Jay Way | 1967 | Magical Mystery Tour | 1967 |
| Can't Buy Me Love | 1964 | Martha My Dear | 1968 |
| Carry That Weight | 1969 | Maxwell's Silver Hammer | 1969 |
| The Continuing Story of Bungalow Bill | 1968 | Mean Mr. Mustard | 1969 |
| Cry Baby Cry | 1968 | Mother Nature's Son | 1968 |
| Dear Prudence | 1968 | Norwegian Wood (This Bird Has Flown) | 1965 |
| Dig a Pony | 1970 | Not a Second Time | 1963 |
| Do You Want to Know a Secret | 1963 | Nowhere Man | 1965 |
| Doctor Robert | 1966 | Ob-La-Di, Ob-La-Da | 1968 |
| Don't Let Me Down | 1969 | Octopus's Garden | 1969 |
| Don't Pass Me By | 1968 | Old Brown Shoe | 1969 |
| Drive My Car | 1965 | One After 909 | 1970 |
| Eight Days a Week | 1964 | Only a Northern Song | 1969 |
| Eleanor Rigby | 1966 | Paperback Writer | 1966 |
| Everybody's Got Something to Hide Except Me and My Monkey | 1968 | Penny Lane | 1967 |
| | | Please Please Me | 1963 |
| Fixing a Hole | 1967 | Polythene Pam | 1969 |
| The Fool on the Hill | 1967 | P.S. I Love You | 1962 |
| From Me to You | 1963 | Revolution 9 | 1968 |
| Glass Onion | 1968 | Rocky Raccoon | 1968 |
| Golden Slumbers | 1969 | Savoy Truffle | 1968 |
| Good Day Sunshine | 1966 | Sexy Sadie | 1968 |
| Good Morning Good Morning | 1967 | Sgt. Pepper's Lonely Hearts Club Band | 1967 |
| Got to Get You into My Life | 1966 | She Came In Through the Bathroom Window | 1969 |
| Happiness Is a Warm Gun | 1968 | She Loves You | 1963 |
| A Hard Day's Night | 1964 | She Said She Said | 1966 |
| Hello, Goodbye | 1967 | She's a Woman | 1964 |
| Helter Skelter | 1968 | She's Leaving Home | 1967 |
| Here Comes the Sun | 1969 | Sie Liebt Dich | 1964 |
| Here, There and Everywhere | 1966 | Strawberry Fields Forever | 1967 |
| Hey Bulldog | 1969 | Things We Said Today | 1964 |
| Hey Jude | 1968 | Ticket to Ride | 1965 |
| Honey Pie | 1968 | Tomorrow Never Knows | 1966 |
| I Am the Walrus | 1967 | We Can Work It Out | 1965 |
| I Don't Want to Spoil the Party | 1964 | When I'm Sixty-Four | 1967 |
| I Me Mine | 1970 | While My Guitar Gently Weeps | 1968 |
| I Saw Her Standing There | 1963 | Why Don't We Do It in the Road? | 1968 |
| I Should Have Known Better | 1964 | Wild Honey Pie | 1968 |
| I Wanna Be Your Man | 1963 | With a Little Help from My Friends | 1967 |
| I Want to Hold Your Hand | 1963 | Within You Without You | 1967 |

*Continued on next page...*

*(Continued from previous page)*

| Song | Year | Song | Year |
|---|---|---|---|
| If I Fell | 1964 | Yellow Submarine | 1966 |
| If I Needed Someone | 1965 | Yer Blues | 1968 |
| I'll Cry Instead | 1964 | You Know My Name (Look Up the Number) | 1970 |
| I'll Follow the Sun | 1964 | You Never Give Me Your Money | 1969 |
| I'm a Loser | 1964 | Your Mother Should Know | 1967 |
| I'm Happy Just to Dance with You | 1964 | You're Going to Lose That Girl | 1965 |
| I'm Looking Through You | 1965 | You've Got to Hide Your Love Away | 1965 |
| I'm Only Sleeping | 1966 | | |
