# Supplementary material for: How science gets by with a little help from the Beatles: Detecting cultural wordplay in scientific literature using large language models
Source: PLoS One. 2026 Sep 9;21(9):e0356147. doi: 10.1371/journal.pone.0356147 (PMC13557405; doi:10.1371/journal.pone.0356147)
Supplement: Supplementary file 2 [file pone.0356147.s002.pdf]

S2 Table. Selected Beatles lyrics and their source song.

Lyric	Song
You say you want a revolution	Revolution
Come together, right now	Come Together
It's getting better all the time	Getting Better
Help! I need somebody	Help!
Love is all you need	All You Need Is Love
Try to see it my way	We Can Work It Out
Will you still need me, will you still feed me	When I'm Sixty-Four
I read the news today, oh boy	A Day in the Life
Something in the way she moves	Something
Get back to where you once belonged	Get Back
Living is easy with eyes closed	Strawberry Fields Forever
Take a sad song and make it better	Hey Jude
You say yes, I say no	Hello, Goodbye
I'd like to be under the sea	Octopus's Garden
Look at all the lonely people	Eleanor Rigby
Nothing's gonna change my world	Across the Universe
Speaking words of wisdom	Let It Be
When I find myself in times of trouble	Let It Be
Let me take you down	Strawberry Fields Forever
Let me tell you how it will be	Taxman
All my troubles seemed so far away	Yesterday
Close your eyes and I'll kiss you	All My Loving
I give her all my love	And I Love Her
I think I'm gonna be sad	Ticket to Ride
In the end, the love you take is equal to the love you make	The End
In the town where I was born	Yellow Submarine
Life is very short	We Can Work It Out
Oh dear, what can I do	Baby's in Black
She was just seventeen	I Saw Her Standing There
Standing on the dock in Southampton	The Ballad of John and Yoko
There are places I remember	In My Life
There's no time for fussing and fighting	We Can Work It Out
We all live in a yellow submarine	Yellow Submarine
You think you lost your love	She Loves You
