# Supplementary material for: How science gets by with a little help from the Beatles: Detecting cultural wordplay in scientific literature using large language models
Source: PLoS One. 2026 Sep 9;21(9):e0356147. doi: 10.1371/journal.pone.0356147 (PMC13557405; doi:10.1371/journal.pone.0356147)
Supplement: Supplementary file 3 [file pone.0356147.s003.pdf]

S1 Appendix. Wordplay detection.

LLM prompts

We evaluate an LLM to automatically detect wordplay at the level of a single reference—a song title or a lyric—passing all of its candidate article titles simultaneously. The classifier is driven by two prompts: a single *system* prompt that fixes the task and the required output format, and a per-reference *user* prompt that supplies the reference, the few-shot examples, and the titles to classify. To make the procedure fully reproducible, we report both the generic templates from which every prompt is instantiated and a concrete worked example.

The same system prompt is used for every reference; the placeholder `<REFERENCE TYPE>` is set to `song title` when classifying song-title candidates and to `lyric` when classifying lyric candidates (Listing 1).

Listing 1. Generic system prompt template for evaluating Beatles wordplay.

```
-----------------------------------------------------------------------------------------
You are a helpful and witty research assistant specialized in language and humor recognition. Your task is to
determine whether each academic article title in the list below is a humorous wordplay on a specific Beatles
<REFERENCE TYPE>. A humorous wordplay means the title creatively references or rephrases the Beatles <REFERENCE
TYPE> in a witty, ironic, or playful way--beyond merely reusing the exact <REFERENCE TYPE>.

You should explain your reasoning in one sentence before giving your final
answer. Your output must be a JSON array where each item has:
    * scopus_id: the unique identifier of the paper,
    * explanation: a one-sentence explanation of your reasoning,
    * answer: either "Yes" or "No" indicating whether the title is a humorous
wordplay on the <REFERENCE TYPE>.

Always prioritize concise, clear, and justified explanations. Only say "Yes" when there's clear humorous intent
or play on words involving the <REFERENCE TYPE>.
-----------------------------------------------------------------------------------------
```

For each reference, the user prompt is built from the fixed JSON template in Listing 2 by filling in the reference, five human-annotated few-shot examples that help the LLM improve its answer quality [1], and all candidate article titles retrieved for that reference. Lyrics are classified with the analogous prompt, supplying the lyric in place of the song title.

Listing 2. Generic user prompt template for evaluating Beatles wordplay.

```
-----------------------------------------------------------------------------------------
{
  "beatles_song": "<REFERENCE>",
  "few_shot_examples": [
    {
      "scopus_id": "<EXAMPLE SCOPUS ID>",
      "paper_title": "<EXAMPLE ARTICLE TITLE>",
      "beatles_song": "<EXAMPLE REFERENCE>",
      "explanation": "<ONE-SENTENCE RATIONALE>",
      "answer": "<Yes or No>"
    },
    ... (five human-annotated examples in total)
  ],
  "titles_to_classify": [
    {
      "scopus_id": "<SCOPUS ID>",
      "title": "<ARTICLE TITLE>"
    },
    ... (all candidate titles retrieved for this reference)
  ]
}
-----------------------------------------------------------------------------------------
```

Listing 3 shows a concrete, truncated instantiation of this template for the song ‘Ticket to Ride’. Listing 4 shows the LLM output for these two article titles. For clarity, we include the full article title again in Listing 4; however, in practice, the outputs contain only the Scopus ID, explanation, and answer in order to reduce API usage costs.

Listing 3. Example user prompt for evaluating Beatles wordplay.

```
-----------------------------------------------------------------------------------------
{
  "beatles_song": "Ticket to Ride",
  "few_shot_examples": [
    {
      "scopus_id": "SCOPUS_ID:84896352755",
      "paper_title": "There will be an answer; let it bleed",
      "beatles_song": "Let It Be",
      "explanation": "The title mimics two exact lines 'There will be an
        answer, let it be' from the song text of 'Let It Be' with a twist
        ('Let it bleed'), which is a clear humorous wordplay.",
      "answer": "Yes"
    },
    {
      "scopus_id": "SCOPUS_ID:33646982325",
      "paper_title": "Everybody's got something to hide except me and my
        patented monkey: patentability of cloned organisms.",
      "beatles_song": "Everybody's Got Something To Hide Except Me and My
        Monkey",
      "explanation": "This is a direct reuse of the song title in the context of
        the paper with light irony, fitting the category of humorous wordplay.",
      "answer": "Yes"
    },
    {
      "scopus_id": "SCOPUS_ID:85194998005",
      "paper_title": "Come for climate, stay for community: Acting, emoting,
        and staying together through the climate crisis",
      "beatles_song": "Come Together",
      "explanation": "It is just a coincidence that the words in the song title
        appear in this article title, it is not a reference to the song.",
      "answer": "No"
    },
    ...
  ],
  "titles_to_classify": [
    {
      "scopus_id": "SCOPUS_ID:85178946808",
      "title": "Pricing Analysis for Railway Multi-Ride Tickets: An
        Optimization Approach for Uncertain Demand within an Agreed Time Limit"
    },
    {
      "scopus_id": "SCOPUS_ID:85062327809",
      "title": "Ticket to a bubble ride: Cargo sorting into exosomes and
        extracellular vesicles"
    },
    ...
  ]
}
-----------------------------------------------------------------------------------------
```

Listing 4. Example LLM response.

```
-----------------------------------------------------------------------------------------
[
  {
      "scopus_id": "SCOPUS_ID:85178946808",
      "title": "Pricing Analysis for Railway Multi-Ride Tickets: An Optimization
        Approach for Uncertain Demand within an Agreed Time Limit",
      "explanation": "The title discusses railway tickets but does not
        creatively reference or play on the Beatles song title 'Ticket to Ride'.",
      "answer": "No"
  },
  {
      "scopus_id": "SCOPUS_ID:85062327809",
      "title": "Ticket to a bubble ride: Cargo sorting into exosomes and
        extracellular vesicles",
      "explanation": "The phrase 'Ticket to a bubble ride' is a playful twist
```

```
        on the original song title, suggesting a humorous context related to
        cargo sorting.",
      "answer": "Yes"
  }
]
-------------------------------------------------------------------------------------
```

References

1. Brown T, Mann B, Ryder N, Subbiah M, Kaplan J, Dhariwal P, et al. Language models are few-shot learners. Advances in Neural Information Processing Systems. 2020;33:1877-901. Available from: https://arxiv.org/abs/2005.14165.
